# Thermal and Mechanochemical Tuning of the Porphyrin Singlet-Triplet Gap for Selective Energy Transfer Processes: A Molecular Dynamics Approach

**DOI:** 10.1021/acs.jctc.1c00291

**Published:** 2021-08-05

**Authors:** Felipe Zapata, Martina Nucci, Obis Castaño, Marco Marazzi, Luis Manuel Frutos

**Affiliations:** ‡ Departamento de Química Analítica, Química Física e Ingeniería Química, Universidad de Alcalá, Ctra. Madrid-Barcelona, Km 33.600, Alcalá de Henares, Madrid E28805, Spain; § Instituto de Investigación Química “Andrés M. del Rio” (IQAR), Universidad de Alcalá, Ctra. Madrid-Barcelona, Km 33.600, Alcalá de Henares, Madrid E-28805, Spain

## Abstract

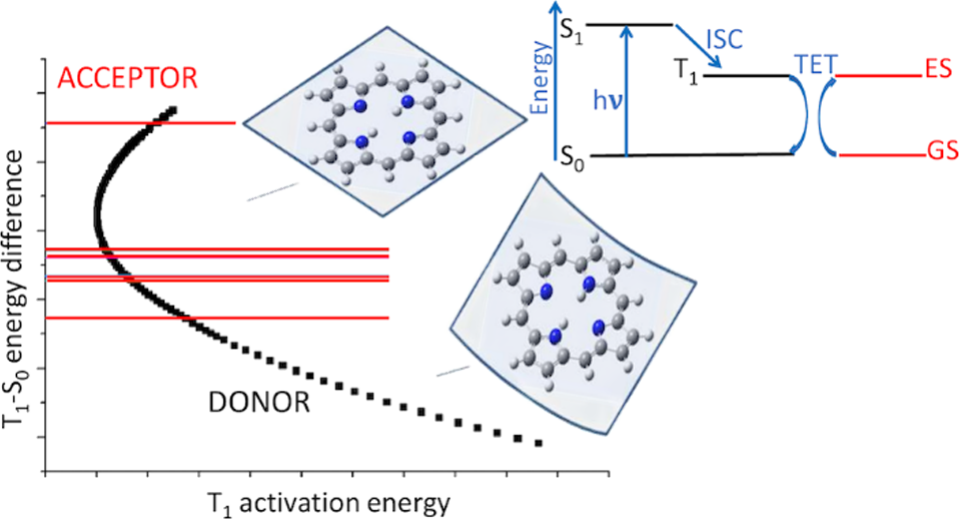

Molecular dynamics simulations provide fundamental knowledge on the reaction mechanism of a given simulated molecular process. Nevertheless, other methodologies based on the “static” exploration of potential energy surfaces are usually employed to firmly provide the reaction coordinate directly related to the reaction mechanism, as is the case in *intrinsic reaction coordinates* for thermally activated reactions. Photoinduced processes in molecular systems can also be studied with these two strategies, as is the case in the triplet energy transfer process. Triplet energy transfer is a fundamental photophysical process in photochemistry and photobiology, being for instance involved in photodynamic therapy, when generating the highly reactive singlet oxygen species. Here, we study the triplet energy transfer process between porphyrin, a prototypical energy transfer donor, and different biologically relevant acceptors, including molecular oxygen, carotenoids, and rhodopsin. The results obtained by means of nanosecond time-scale molecular dynamics simulations are compared to the “static” determination of the reaction coordinate for such a thermal process, leading to the distortions determining an effective energy transfer. This knowledge was finally applied to propose porphyrin derivatives for producing the required structural modifications in order to tune their singlet-triplet energy gap, thus introducing a mechanochemical description of the mechanism.

## Introduction

Molecular dynamics is a fundamental tool for shedding light on many processes ranging from biological to chemical systems. In spite of ignoring quantum effects, classical dynamics within the Born–Oppenheimer approximation provides faithful results for many systems and conditions.^1,2^ The reliability of the result of the classical dynamics depends on the accuracy of the potential for describing interactions among the particles of the system;^3,4^ therefore, the use of accurate potential energy surfaces (PES) is of central importance, providing molecular forces, which describe accurately the interactions in the molecular systems in an affordable computational time. There have been different approaches for determining forces in molecular dynamics: (i) classical force fields describing conformational changes,^5−7^ (ii) reactive force fields mainly devoted to the optimization of material properties,^8^ (iii) ab initio “on the fly” dynamics (i.e., calculating molecular forces at each trajectory step),^9,10^ and (iv) approximated PES through the interpolation or fitting of energies calculated for a set of structures at a high level of theory, adjusted to minimize the error, as the reproducing kernel Hilbert space (RKHS),^11^ neural networks,^12^ interpolating moving least squares,^13^ modified Shepard interpolation,^14^ and gradient-enhanced kriging (GEK).^15^ All such strategies have positive sides and drawbacks, depending on the size of the system to be studied, the required simulation time, and the quality of the predictions.^1,16,17^

An alternative to these methodologies, which has been followed in this work, is the construction of analytical PES. It is possible to construct these analytical functions by spanning the PES as an extrapolation from some key configurations like the Frank–Condon (FC) structure and, if present, other nonglobal minima. The advantages of this methodology are the fast evaluation of the molecular forces for every structure along the dynamics, hence making it possible to reach large simulation times without losing accuracy in the force prediction as long as the expansion is correct in the configuration space covered by the dynamics simulation. Moreover, this approximation can be also applied to force fields for electronic excited states, paving the way to the description of any photophysical phenomenon. The simplest way to construct the analytical PES from local approximations is to use a second-order energy derivative expansion for all the electronic states under study. This kind of simple approach can be extremely useful when the molecular system presents a well-defined global minimum in the electronic state populated by the molecule (e.g., S_0_) and the other states of interest are well represented by up to a second-order approach (e.g., S_1_, if our aim is to simulate the S_0_ → S_1_ absorption spectrum).

In this study we will focus on the triplet energy transfer (TET) process involving a triplet donor (^3^D) and a singlet (^1^A) or triplet (^3^A) acceptor that when reaches a close contact can fulfill the energy transfer criteria. The contact between both molecules permits interchanging their total spin momentum by an electron-exchange mechanism (i.e., Dexter-type energy transfer), making the electronic spin multiplicity of the whole system (D and A) conserved and making the process spin-allowed.^18,19^ As it has been discussed elsewhere, the TET rate constant depends mainly on three factors: the diffusion rate constant of A and D in the solvent, the efficient overlap of the wavefunctions of both molecules, and the singlet-triplet (S_0_-T_1_) energy gap in both donor (*E*_T_^D^) and acceptor (*E*_T_^A^) molecules.^20−24^ Nevertheless TET can also take place as an intramolecular process where the contact between donor and acceptor moieties of the molecule is mediated by linkers.^25,26^

The maximum TET efficiency is reached when the resonance condition is fulfilled, i.e., when *E*_T_^D^ = *E*_T_^A^. As the difference between the two gaps, defined as Δ*E*_T_ = *E*_T_^A^ – *E*_T_^D^, becomes positive (i.e., the donor S_0_-T_1_ energy gap is lower than the corresponding acceptor gap, *E*_T_^D^ < *E*_T_^A^), the rate constant of the process decays exponentially within 3–4 kcal/mol.^23^ In the case of exothermic reactions, i.e., *E*_T_^D^ > *E*_T_^A^, the rate constant of the energy transfer in the condensed phase matches the rate constant of the D-A encounter probability, and there is no experimental evidence of decreasing quenching efficiency as the reactions becomes even more exothermic.^27^

**Figure 1:**
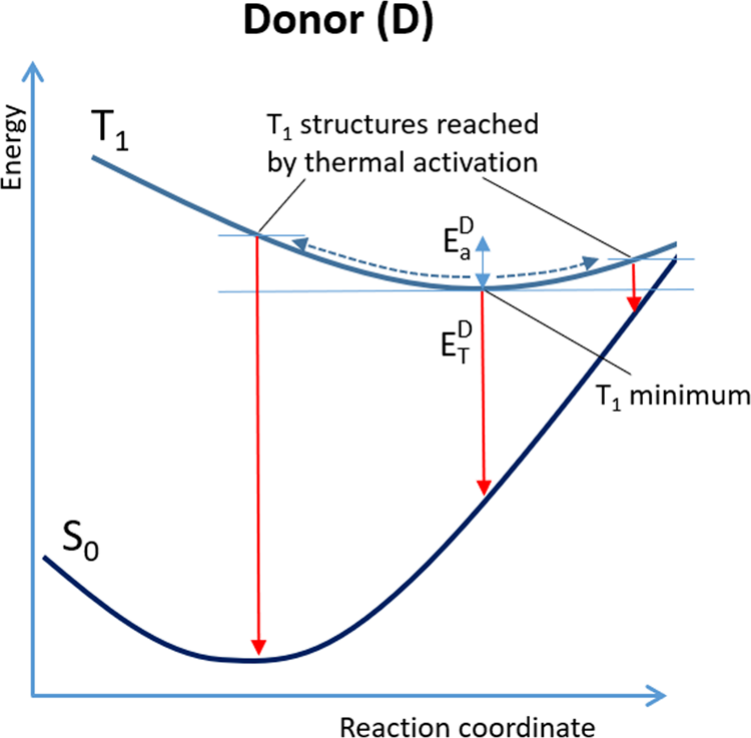
Energy diagram showing how the donor T_1_ activation energy (*E*_a_^D^) can modify the donor T_1_-S_0_ energy difference (*E*_T_^D^), thus modifying the energy criterion to meet the resonance condition with the acceptor.

The modulation of the donor S_0_-T_1_ energy gap (*E*_T_^D^) is therefore an ambitious and highly relevant target, since it can be chosen in order to optimize the TET efficiency of any specific mechanism. As we have previously demonstrated,^28^ the modulation of *E*_T_^D^ can be determined as a function of the activation energy (i.e., the thermal energy necessary to achieve a given variation of the T_1_ energy referred to as the T_1_ minimum structure), providing a reaction coordinate for the process (see Figure 1).

Here, we choose porphyrin as a triplet donor while taking into account different relevant acceptors: molecular oxygen, a series of carotenoids (lutein, zeaxanthin, β-carotene, and lycopene), and the retinal visual pigment.

**Figure 2:**
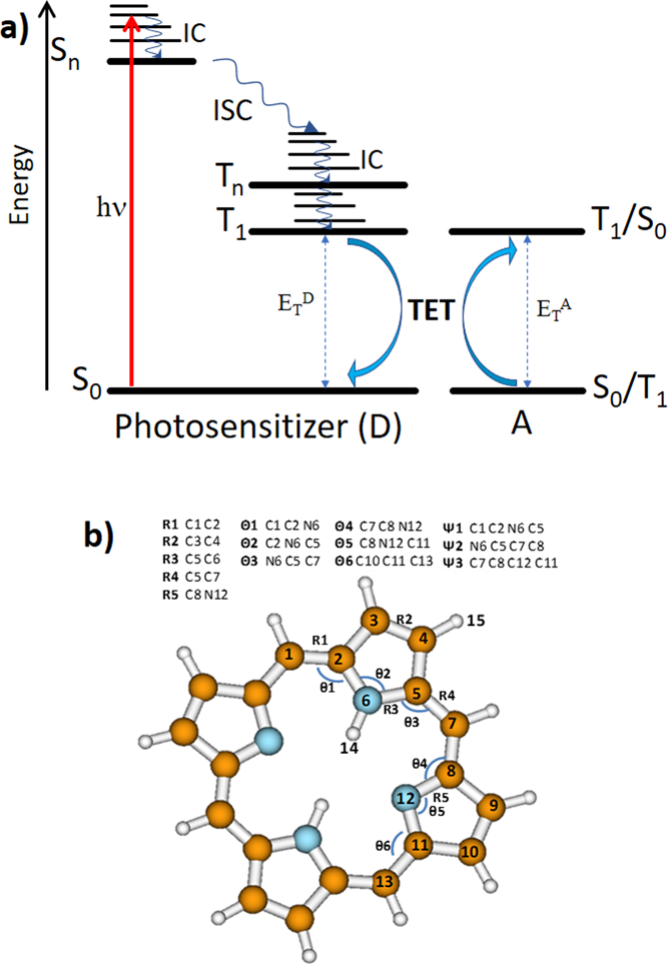
(a) Scheme showing the photophysics underlying photosensitizer light absorption: the singlet excited manifold is directly populated by a vertical transition followed by internal conversion (IC) and intersystem crossing (ISC) to the triplet manifold. Finally, the lowest triplet state (T_1_) can be populated, from where a TET process can be activated between the photosensitizer, i.e., the donor (D), and an acceptor (A); in the case shown, the singlet-triplet energy gap of D and A indicate the resonance condition (*E*_T_^D^ = *E*_T_^A^). (b) Structure of the donor considered in this study (porphyrin), including the internal coordinates that were found to be more involved in modulating the TET efficiency. These internal coordinates are shown on two pyrrole rings. By symmetry, the same internal coordinates do apply for the specular two pyrrole rings.

Especially, the TET between triplet porphyrin (a paradigmatic photosensitizer)^29^ and triplet molecular oxygen ^3^O_2_ (X^3^Σ^–^_g_) to generate singlet oxygen is a model system for the study of photodynamic therapy (PDT) treatments:^30^ After light absorption promotes the spin-allowed population of the singlet excited state manifold of the photosensitizer (S*_n_*, usually with *n* > 1), followed by a fast vibrational decay to S_1_ and intersystem crossing to populate the triplet manifold, finally assuring that most of the incoming photon energy populates T_1_, from where TET can take place (Figure 2a).

We should note that other acceptors were also found to be good triplet acceptors (e.g., cyclooctatetraene can accept energy donated by porphyrin^31^), although less relevant from the biological point of view.

On the other hand, carotenoid polyenes have also attracted attention as they were suggested to provide photoprotection to photosynthetic pathways by quenching the chlorophyll (containing a porphyrin derivative chromophore) triplet state through TET, hence avoiding the eventual singlet oxygen formation from chlorophyll.^32^

The retinal visual pigment was also taken into consideration embedded in its opsin protein, constituting a Schiff-base possibly acting as a triplet acceptor for night vision.^33−35^ Moreover, retinal–opsin complexes (especially rhodopsin) constitute also an intriguing case of endothermic TET process when porphyrin is the triplet donor, while in all other cases (molecular oxygen and carotenoids) a more common exothermic TET process is expected.

After constructing T_1_ analytical PES for porphyrin, we perform molecular dynamics and analyze the TET reaction coordinate, to understand which normal modes are mainly involved in the modulation of the singlet-triplet energy gap. This knowledge is finally applied to the molecular design of porphyrin derivatives, by introducing a mechanical strain intended to activate the desired normal modes, showing that a modulation of *E*_T_^D^ can be effectively achieved, especially for exothermic TET processes.

## Computational Methods

The minimum energy structures of porphyrin in the lowest singlet (S_0_) and triplet (T_1_) states were determined by applying density functional theory (DFT) by using, after comparing different functionals (Table S1), the hybrid functional B3LYP^36,37^ with a CC-pVDZ basis set.

First and second derivatives of the energy in Cartesian coordinates were determined for the optimized structures in both electronic states. All the calculations were performed with the Gaussian09 software package.^38^ The complete molecular dynamics simulations within the canonical ensemble were performed using our own code, as we discuss in the following section.^39^

The triplet energy reaction coordinate for the donor has been obtained by using the algorithm, and the definition is described elsewhere^21,28^ and briefly explained below, using analytical gradients determined with the electronic methods described above. More in detail, the triplet energy reaction coordinate (***q***^RC^) defines the minimum energy configuration of the donor on its PES fulfilling a certain resonance condition imposed by the energy of the acceptor. We should highlight that the method employed allows us to treat D and A molecules separately; hence, it is not necessary to study the D···A supermolecule. The positive side of this approach is that we can avoid studying the details of the D···A interactions. Nevertheless, the drawback is that we cannot consider the overlap between A and D wavefunctions, thus solely relying on the TET resonance energy criterium.

Moreover, in flexible donors, it is possible that nonvertical endothermic energy transfer can play a role, due to a large reduction of *E*_T_^D^ induced by geometrical distortions, which require low activation energy (see Figure 1). Hence, a geometrical distortion parameter (γ*_i_*) can be defined for each internal coordinate *i* as follows:
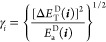
where Δ*E*_T_^D^ is the difference between the S_0_-T_1_ energy of the distorted donor and that of the donor in its T_1_ minimum.

For carotenoids, the same level of theory used for porphyrin was applied. Especially, the carotenoid structures were optimized in S_0_ followed by single point evaluation of the T_1_ energy.

The T_1_-S_0_ energy gap of retinal in rhodopsin was calculated elsewhere through the CASPT2//CASSCF approach,^33^ while the T_1_-S_0_ energy gap of molecular oxygen was calculated here at the CASPT2 level, as shown in the Supporting Information (Figures S1–S3). All CASPT2 and CASSCF calculations were performed with the OpenMolcas code.^40^

We should note that both retinal and molecular oxygen are treated at a more sophisticated ab initio approach due to the intrinsic multiconfigurational nature of their electronic transition. This does not apply to carotenoids and porphyrin, which were shown to be correctly described by DFT methods. Moreover, the large size of these molecules and of their relative active space makes their multiconfigurational treatment unfeasible.

Porphyrin phosphorescence and molecular oxygen absorption were simulated at different temperatures (50 and 300 K), by applying the Boltzmann distribution, to highlight how temperature affects their possible superposition and thus the energy transfer probability (Figures S4 and S5).

Finally, different mechanochemical strains were induced on porphyrin with two different strategies: (i) by including a series of linkers connected to opposite pyrrole rings (−O(CH_2_)*_n_*O–, with *n* = 5, 7, and 9) and optimizing their geometry and (ii) performing a relaxed scan along the distance connecting the two carbon atoms where the linkers are anchored (steps of 0.1 Å).

In all cases, all molecules were studied in vacuum. To justify the choice of the B3LYP functional, additional functionals were tested (see the Supporting Information). All calculations related to mechanochemistry were performed at the B3LYP/CC-pVDZ level of theory, in order to be comparable with the unsubstituted porphyrin.

## Methodology Development

TET involves two different states of a donor–acceptor complex, the state before the energy transfer [*D···A] and after the transfer [D···*A], where the asterisk denotes the excitation. It has been proposed elsewhere that the TET process can be separated in terms of single transitions *D → D and A → *A in the very weak coupling limit,^21^ as usually occurs in solution. Following this approach, in order determine the excitation transfer rate it is necessary to know the energy of each electronic transition and study whether the resonance condition (i.e., same electronic transition energy for both molecules) can be fulfilled. Even if the energy of the vertical transitions for *D → D and A → *A does not match, the resonance condition can be reached by thermal activation. Implying that energy resonance is fulfilled following a minimum energy coordinate (so called the TET coordinate), the activation energy for a given pair of donor–acceptor can be determined.^21^ On the other hand, for the study of the TET from a dynamical description of the system, it is in principle necessary to use accurate force fields for both D and A in the ground and excited states. Nevertheless, here, we focus the attention on the dynamic description of *D → D, while relying on static calculations for obtaining the A → *A energies.

### Construction of Analytical PES in Internal Coordinates

In order to construct the PES of porphyrin in a given electronic state we have performed a quadratic expansion of the potential energy function in terms of internal coordinates:
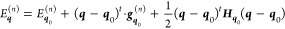
1where ***q*** is a vector in internal coordinates denoting any configuration (i.e., molecular structure), and ***q*_0_** is also a vector in internal coordinates corresponding to the reference configuration for the expansion, in this case corresponding to the Franck–Condon structure. ***g***_***q***_0__^(*n*)^ and ***H***_***q***_0__ are the energy gradient vector and Hessian matrix of the (*n*) electronic state evaluated for the ***q*_0_** geometry, both expressed in internal coordinates. From the approximate PES given in (eq 1), the energy gradient vector obtained is
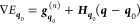
2

The election of internal instead of Cartesian coordinates for deriving the force field has some advantages, since the curvilinear coordinate system, if correctly chosen, preserves the quadratic approximation more accurately, making the spanned PES more precise as the displacement vector (***q – q***_0_) increases.^41^

It is therefore necessary to select a set of internal coordinates for the expansion of PES by following a certain chemical knowledge, in order to accurately predict the energy of the extrapolated points. Since, usually, first and second derivatives are available in Cartesian coordinates, it is necessary to transform the Cartesian derivatives into internal derivatives. The relation between the derivatives of the energy with respect to internal and Cartesian coordinates is described by the Wilson ***B*** matrix whose elements  are given by the derivatives of the internal coordinates with respect to the Cartesian coordinates.^42^ Using this matrix, the relation between the gradients in Cartesian and internal coordinates are given by:
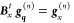
3

and
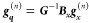
4where ***g***_*x*_^(*n*)^ is the energy gradient vector in Cartesian coordinates evaluated for an arbitrary ***X*** configuration, expressed in Cartesian coordinates. Since the ***B*** matrix is not squared, its inversion requires to find the generalized inverse of a ***G*** matrix given by ***G*** = ***BUB****^t^* where ***U*** is a unitary matrix. In the case of using a set of redundant internal coordinates, the inversion of the ***G*** matrix requires a previous diagonalization followed by the elimination of the zero eigenvalues resulting from the redundant internal coordinates, keeping only those eigenvectors corresponding with the 3N-6 degrees of vibrational freedom.^43^ The relation between the Hessian matrices in both set of coordinates is obtained after differentiating (eq 3) and (eq 4):
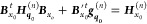
5

and
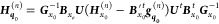
6where ***H***_*x*0_^(*n*)^ is the Hessian matrix in Cartesian coordinates evaluated for the ***X*_0_** configuration, and ***B*′** is a three-dimensional array whose elements  are the second derivatives of the *i^th^* internal coordinate with respect to the *j*th and *k*th Cartesian coordinates.

In order to obtain the numerical values of the gradient and Hessian matrix in internal coordinates, analytical expressions were developed and implemented for the first and second derivatives of the internal coordinates, with respect to Cartesian coordinates (i.e., *B_ij_* and *B_ijk_* terms).

### Molecular Dynamics

In order to simulate the molecular system at constant temperature (i.e., an NVT canonical ensemble), the Nosé–Hoover chain of thermostats and the equations resulting from the extended-Lagrangian method were applied, thus generating non-Hamiltonian dynamics.^44^ The equations of motion were integrated using a time-reversible integrator, using the Liouville approach through the Trotter factorization.^45^ As it has been shown, a chain of thermostats can drive the variables of the harmonic oscillator to a canonical distribution,^46^ and since the potential developed in this work is given by a collection of harmonic oscillators, the Nosé–Hoover chain of thermostats was considered an appropriate selection.

The initial conditions for simulating the canonical ensemble are prepared by obtaining random velocities of the Maxwell–Boltzmann distribution, assuming that every component of the velocity of each atom can be considered as an independent Gaussian random variable.^5^ In order to avoid misinterpretation and numerical drifting in the temperature, the motion and rotation of the center of mass is removed at every step of integration in both ensembles.

The dynamic simulations were carried out in the gas phase at 300, 200, 100, and 50 K. The simulation time was 1.0 ns using an integration step of 0.1 fs.

The conservation of the total energy was checked by the variance in the temperature, given by the following equation:
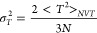
7where *N* is the number of atoms.^3,47^

The full code can be accessed at the following repository: https://github.com/resmol/dynamics

## Results and Discussion

### Dynamics of the Triplet Porphyrin Sensitization of Triplet Oxygen

Using the methodology described above, we have studied the dynamic behavior of porphyrin in the triplet (T_1_) state and the evolution of the T_1_-S_0_ energy gap along the simulation.

**Figure 3:**
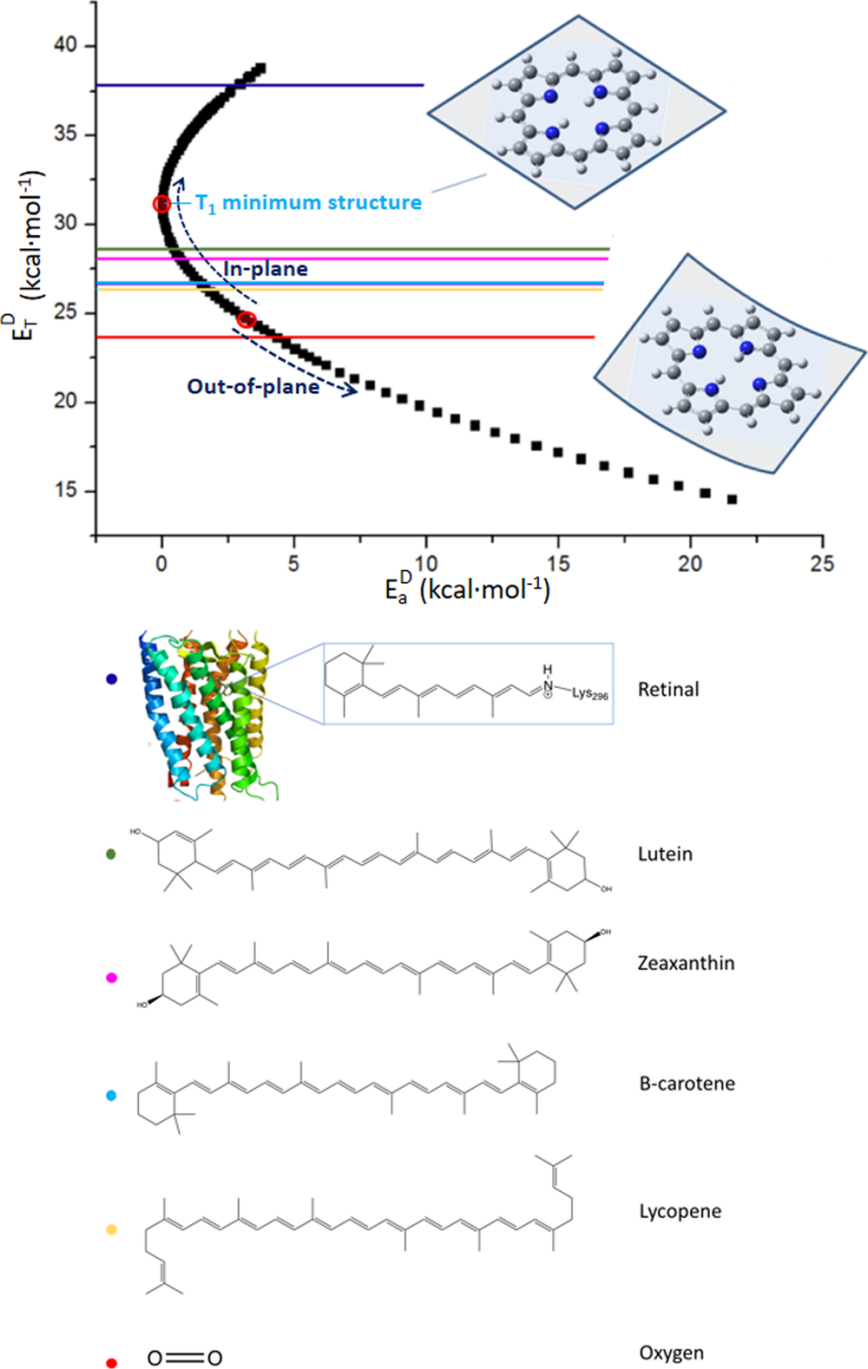
T_1_-S_0_ energy gap, *E*_T_^D^, as a function of the activation energy on T_1_, *E*_a_^D^, (black), obtained from the determination of the TET reaction coordinate for triplet porphyrin (T_1_ → S_0_ transition). Out-of-plane distortions are active below 24 kcal·mol^–1^, while in-plane distortions play the key role above 24 kcal·mol^–1^. All acceptor S_0_-T_1_ gaps are also indicated with a colored line, displaying the corresponding structure at the bottom.

Dynamics simulation provides, among other information, the probability distribution for the configuration space, allowing us to identify the frequency (or probability density) of a given range of coordinate values in a given electronic state for certain conditions (temperature, etc.). Different electronic properties depending on the configuration of the system can be determined and expressed in terms of probability density. This is the case of the energy gap between two electronic states. For the study of TET, it is necessary to measure the T_1_-S_0_ energy gap of porphyrin along the trajectory, obtaining from a large simulation time a distribution function of the T_1_-S_0_ energy gap. This distribution can be directly related to the T_1_ → S_0_ emission (phosphorescence) spectrum of porphyrin. For the T_1_ minimum energy structure, the energy of the T_1_-S_0_ transition is 31 kcal·mol^–1^. This value corresponds to a T_1_ activation energy equal to zero (see Figure 3). At the same time, the T_1_-S_0_ energy gap of the selected acceptors spans a wide energy window, from ca. 23 kcal·mol^–1^ for triplet molecular oxygen to ca. 38 kcal·mol^–1^ for retinal as the chromophore of bovine rhodopsin. All carotenoids lie in between, with a S_0_-T_1_ energy gap of ca. 27 kcal·mol^–1^ for lycopene and β-carotene and around 28 kcal·mol^–1^ for zeaxanthin and lutein. It has to be reminded that while molecular oxygen is a T_1_ species in the ground state, all other studied acceptors are S_0_ ground-state species (Figure 2a).

From a chemical point of view, there is not an easy way to predict how every porphyrin internal coordinate can stabilize or destabilize both T_1_ and S_0_ states in order to modulate the TET process. In the following study, we show how dynamics simulations at constant temperature provide realistic geometries that, coupled to the determination of the TET reaction coordinate, identify the molecular distortions having a greater statistical weight in the TET process.

Therefore, the geometrical distortion parameters γ*_i_*, describing the TET reaction coordinate,^28^ was calculated for every geometry obtained in each integration step along the trajectories. Figure 2b shows the internal coordinates that have the greatest influence in the TET process between porphyrin and any donor, for constant temperature simulation at 300 K. Correspondingly, Table 1 reports the values of γ*_i_* for the set of internal coordinates shown in Figure 2b in the range of T_1_-S_0_ energy gaps of 23–38 kcal/mol. According to these values, the out-of-plane torsions are the most feasible geometrical distortions at room temperature, due to the associated lower force constants (higher amplitude of the vibrational modes) compared to changes in bonds or angle values. Nevertheless, for the same reason, small changes of bonds or angles could account for significant changes in the T_1_ energy, hence resulting in a significant modulation of the TET process.

**Table 1:** Values of the Geometrical Distortion Parameters (γ*_i_*) for the Most Relevant Internal Coordinates of Porphyrin (See Nomenclature in Figure 2b)

coordinate	γi (kcal/mol)	coordinate	γi (kcal/mol)
R1	2.8	Θ3	10.9
R2	2.0	Θ4	10.6
R3	1.6	Θ5	3.6
R4	3.5	Θ6	5.3
R5	1.9	Ψ1	0.8
Θ1	4.2	Ψ2	2.0
Θ2	5.3	Ψ3	1.0

### TET Reaction Coordinate for the Porphyrin/Acceptor System

Singlet oxygen generation by triplet porphyrin takes place according to:
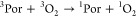


All other acceptors studied here do imply a singlet-to-triplet spin change:
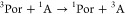


In any case, all involved transitions ^3^O_2_ → ^1^O_2_, ^1^A → ^3^A and ^3^Por → ^1^ Por are spin-forbidden. Nevertheless, as it has been discussed above, the whole process involving both electronic transitions (i.e., D and A taken as a “supermolecule”) are spin-allowed.^43^ On the other hand, both transitions must fulfill the energy resonance condition, which ensures the balance between the transferred and the accepted energy. The triplet energy of the ^3^Por → ^1^Por transition defined as vertical excitation from the ^3^Por relaxed structure (i.e., the T_1_ minimum corresponding to the phosphorescence maximum) is 31 kcal·mol^–1^, while the ^3^O_2_ → ^1^O_2_ transition is, following the same definition, ca. 23 kcal·mol^–1^,^48^ implying that some changes must take place on both molecules in order to reach the resonance condition.^28^ The same situation applies for the carotenoid structures studied here (27–28 kcal·mol^–1^), although resulting in a less exothermic TET process. If we take into consideration the retinal chromophore embedded in the opsin cavity to form rhodopsin, the singlet-triplet energy gap is even larger than porphyrin (38 kcal·mol^–1^), making a less-probable endothermic TET necessary.

In the case of weak coupling limits, since the absolute value of the electronic coupling term is very low compared with other energies involved in the transfer step, the PES of the complex Por···A can be separated in two uncoupled PES for the donor and acceptor partners,^21^ and therefore, the role of the internal coordinates of each molecule in reaching the energy resonance can be analyzed independently. If the resonance condition is assumed to take place with minimum activation energy, the TET reaction coordinate can be determined as described elsewhere.^28^ In Figure 3, the variation of the ^3^Por → ^1^Por transition energy is displayed as a function of the activation energy.

On the other hand, all singlet-triplet transitions of the various acceptors are indicated as corresponding to their ground-state minima structures. Hence, the intersection between the curve representing the ^3^Por → ^1^Por energy gap variation (Figure 3, black) and the line representing the singlet-triplet energy gap of a certain acceptor corresponds to the fulfillment of the resonance condition. This means that we are focusing at finding the minimum energy distortion of porphyrin that would maximize the TET process for a given acceptor. For instance, the energy resonance condition for both D and A transitions (*E*_T_^D^ = *E*_T_^A^) is fulfilled for a porphyrin structure where stretching modes and especially out-of-plane distortions contribute significantly in lowering the S_0_-T_1_ energy gap to equal the S_0_-T_1_ gap of molecular oxygen. In addition, ^3^O_2_ has a very reduced capacity in modulating its S_0_-T_1_ energy gap because of the single stretching mode of the molecule. To demonstrate this assumption, we have calculated the same curve (S_0_-T_1_ energy gap as a function of the activation energy) for ^3^O_2_, showing the much narrower capabilities to modulate the S_0_-T_1_ gap (see the Supporting Information).

In any case, porphyrin shows a significant ability to modulate the ^3^Por → ^1^Por energy gap by thermal fluctuations following the softer modes. In fact, in order to decrease the energy gap, the optimal molecular distortions correspond to in-plane distortions (i.e., stretching modes) for the initial 7 kcal·mol^–1^ (i.e., from 31 to 24 kcal·mol^–1^), while further reduction of this gap is efficiently reached by out-of-plane distortions (sheet-bend-like distortion, see Figure 5), which provides the optimal distortion in order to reach the ca. 23 kcal·mol^–1^ necessary to achieve resonance with the oxygen S_0_-T_1_ gap (^3^Σ_g_ → ^1^Δ_g_ transition). In principle, the larger the out-of-plane distortion (i.e., the more pronounced the sheet-bending), the lower S_0_-T_1_ can be reached, fulfilling, if required, highly exothermic TET processes. Such distortion does not modify the nature of the T_1_ state, as evidenced by energy and spin density considerations (see Figures S7 and S8).

This result could be partially envisioned by looking at the internal coordinates more involved in modulating the TET efficiency (Figure 2b): stretching modes across pyrrole rings (R_1_-to-R_5_) and a couple of torsion modes (Θ_1,2_) on two facing pyrrole rings, responsible for the sheet bending. Indeed, the TET reaction coordinate shown in Figure 3 is composed of the most efficient coordinates in controlling the TET process.

Concerning the other proposed acceptors, all of them fall into the in-plane distortion region. Especially, the carotenoids are the least energy-demanding compounds (3–5 kcal·mol^–1^) still corresponding to a more probable exothermic TET process. This explains well why carotenoids (in their different flavors) were particularly selected by nature to accept the triplet energy from chlorophyll (a porphyrin derivative) in photosynthetic systems, thus preventing damages to this fundamental natural process.

On the other hand, a less feasible endothermic TET process (7 kcal·mol^–1^) is required to photosensitize the retinal pigment and allow vision under dim-light (i.e., infrared) conditions, as experienced by some deep-sea fishes in the presence of chlorophyll^49−53^ and by some patients treated with photodynamic therapy drugs based on porphyrin derivatives.^35^ Although less probable, we show that activation of in-plane stretching modes can make possible the fulfillment of the resonance condition also in this case.

### Estimated Experimental TET Rate Constants

The experimental TET rate constants between a donor and an acceptor in a given solvent can be estimated based on the mechanism involving diffusive and energy transfer steps.^20^
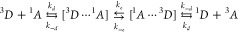
8where *k_d_* and *k*_–*d*_ are the diffusion constants, and *k_e_* and *k*_–*e*_ are the forward and backward energy transfer processes. Taking into account that backward energy transfer is negligible in the case of the endothermic energy transfer process (i.e., the triplet energy of the donor is larger than the triplet energy of the acceptor, hence *k_e_* ≫ *k*_–*e*_), the rate experimental constant can be approximated to:
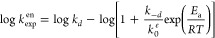
9where the *k*_–*d*_/*k*_0_^*e*^ ratio can only be estimated (*k*_–*d*_/*k*_0_^*e*^ ∼ 0.25) and the activation energy (*E*_a_) can be fitted to a quadratic function:
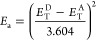
10where kcal/mol units are used for energy. Here, the denominator is equal to , which corresponds to the geometrical distortion parameter^20^ measuring the nonvertical behavior of the donor/acceptor (for comparison, the strongly nonvertical acceptor cyclooctatetraene has a value of 13.5 ).^20^

The experimental energy transfer rate constant is therefore:
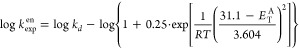
11

Taking into account that the diffusion constant is in the range (depending on the specific experimental conditions) 10^10^ to 10^11^ s^–1^, the estimated experimental energy transfer rate constant (*k*_exp_^en^) is:
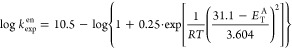
12

**Figure 4:**
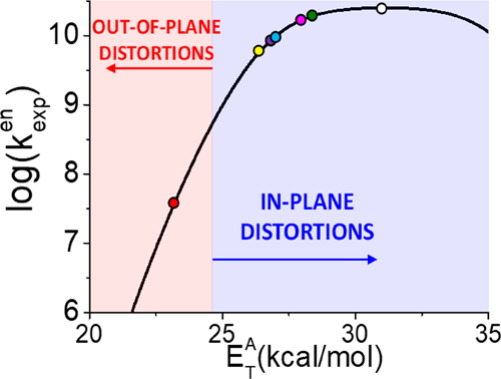
Estimated experimental energy transfer rate constants as a function of the triplet energy of the acceptor molecule, i.e., porphyrin at a temperature of 298 K. The dots indicate the approximate rate constants for the different studied acceptors, when considering only forward energy transfer, i.e., exothermic conditions (color code taken from Figure 3, with the white dot indicating the porphyrin T_1_ minimum structure).

The representation of log*k*_exp_^en^ as a function of the T_1_-S_0_ energy gap (i.e., *E*_T_^A^) for porphyrin is given in Figure 4.

### Comparison of the TET Reaction Coordinate and Dynamics Simulations

**Figure 5:**
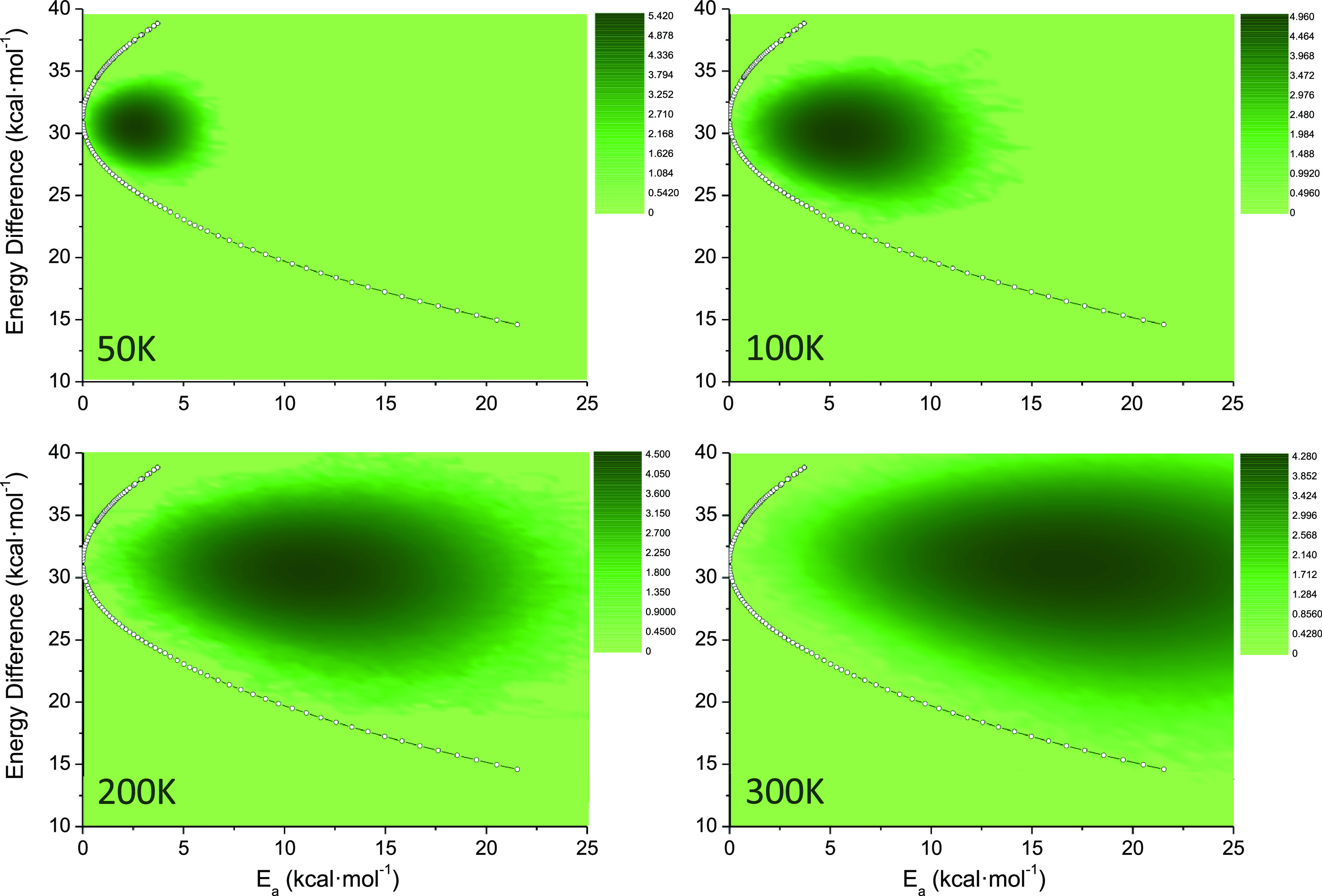
Porphyrin population density obtained from 1 ns molecular dynamics simulations at different temperatures (50, 100, 200, and 300 K) represented as a function of both S_0_-T_1_ energy difference and T_1_ activation energy (density populations are given in a logarithmic scale).

Molecular dynamics of the triplet state porphyrin provides useful information for understanding the feasibility of the TET reaction coordinate. By analyzing the 10^6^ points obtained from the different constant temperature simulations (*T* = 50, 100, 200, and 300 K), the population density can be obtained as a function of the variation of both the S_0_-T_1_ energy gap and T_1_ activation energy (see Figure 5). As expected, porphyrin tends to explore regions with larger variations of the S_0_-T_1_ energy gap as the thermal energy increases, hence allowing the system to reach higher activation energies. This means that, although not selectively, the resonance condition for the TET process to any acceptor can be thermally induced. Indeed, the density distribution is always located within the region defined by the “static” definition of the TET reaction coordinate. As a matter of fact, it would be possible to define essentially the same TET reaction coordinate from molecular dynamics results by simply taking the lowest activation energy structure for any given interval of the S_0_-T_1_ energy gap.

Moreover, from the molecular dynamics results, it is also possible to estimate the fraction of porphyrin molecules that, along the simulation time, reaches the resonance condition to donate energy to a given acceptor. This fraction, which is a function of the selected temperature, can be computed as the overlap between the density populations of the singlet-triplet transitions for both porphyrin and the acceptor. By computing such a population overlap, it is found that the TET rate constant increases exponentially with temperature, as it is predicted by assuming a Boltzmann distribution of the initial states of the donor and acceptor and using the transition state theory.^20^

Alternatively, the TET rate constant can be obtained through the Fermi golden rule,^54^ by integrating the spectral overlap between the normalized spectra related to donor and acceptor singlet-triplet transitions. Such normalized spectra can be extracted from the distribution functions of the S_0_-T_1_ energy gap, available by treating the dynamics data.

Here, we take into consideration the overlap between porphyrin phosphorescence and triplet oxygen absorption, showing the thermal activation of this photosensitization process: at 50 K there is no overlap, while at 300 K a non-negligible, although small, overlap does appear (see Figures S4 and S5), indicating that there is room for improvement in terms of photosensitization efficiency. Nevertheless, most of the biological and biomedical applications related to TET processes are not applicable for temperatures higher than 300 K; hence, porphyrin could be chemically modified in order to match the desired TET resonance condition.

### Induced Mechanochemical Strain

**Figure 6:**
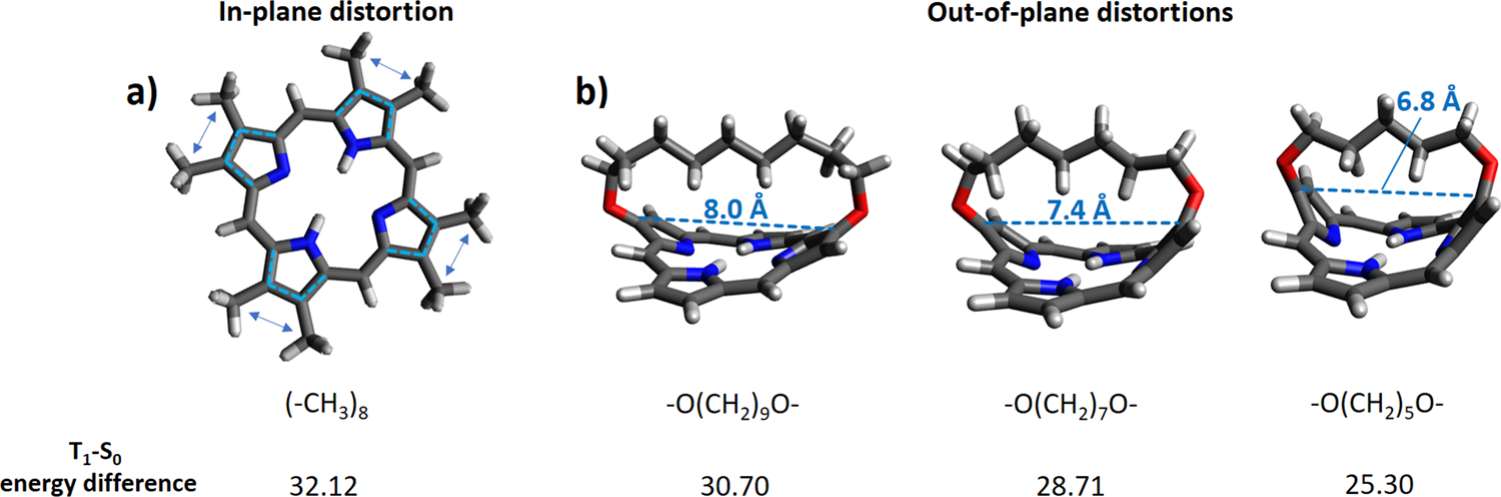
In-plane (a) and out-of-plane (b) distortions induced on porphyrin through suggested substitution patterns. The optimized geometries and the relative T_1_-S_0_ energy difference (in kcal/mol) are shown. The most affected molecular coordinates are shown by dashed lines and arrows.

As we have shown that, in principle, thermal energy could be used to selectively activate a certain T_1_-S_0_ energy gap, we have adopted a chemical substitution strategy in order to propose modified porphyrins matching the expected distortions (see Figure 3). We note that this approach was very recently applied to modify the triplet energy of cyclooctatetraene as the acceptor.^55^ Especially, we do expect that out-of-plane distortions should lower the T_1_-S_0_ energy difference, compared to in-plane distortions, due to differently applied forces induced by a chosen substitution pattern (i.e., mechanochemistry). Especially, we have picked dioxy-alkyl chains of different length to connect two facing pyrrole units, thus reproducing the expected out-of-plane distortion (see Figure 6b). Dioxy-alkyl chains were selected since they do not modify the “original” porphyrin chromophore, and, at the same time, they can be successfully anchored to organic chromophores, as it was previously found by some of the authors in both theoretical and experimental points of view, leading to highly distorted structures of stilbene^56^ and pyrene.^57^

The results (Figure 6b) show that lowering the length of the linker chain, by decreasing the number of methylene groups from nine to five, corresponds to a higher porphyrin distortion and thus to a progressive lowering of the T_1_-S_0_ energy difference, as expected. This trend was also confirmed by performing a relaxed scan along the same anchoring distance (see Figure S6 for details), although we are aware that, from a synthetic point of view, the most distorted structures could be hardly synthesized.

On the other hand, in-plane distortions are more difficult to be implemented, due to the fact that the position and the type of substituent can easily induce out-of-plane distortions as well. Nevertheless, we have found a possible substitution pattern by introducing symmetrically eight methyl groups, two on each pyrrole ring. In this way, an in-plane force is generated due to steric strain between the two −CH_3_ groups on each pyrrole ring, slightly modifying the overall conjugation pattern, and resulting in an increase in the T_1_-S_0_ energy difference compared to both out-of-plane distorted structures and unsubstituted porphyrin (Figure 6a).

## Conclusions

A methodology was developed and implemented for building analytical PES as functions of the first and second derivatives of energy with respect to the internal coordinates. This methodology was used to build the relevant PES (T_1_ and S_0_) involved in the energy transfer processes of a prototypical donor: porphyrin. These analytical PES were used for molecular dynamics simulation at constant temperature (from 50 to 300 K) for the simulation, within the weak coupling limit, of the TET process between triplet porphyrin and different acceptors: molecular oxygen, various types of carotenoids, and rhodopsin. The obtained results indicate that the out-of-plane sheet-bending-like motion and in-plane stretching are the coordinates enabling the process. These structural deformations allow the modulation of the porphyrin T_1_-S_0_ energy gap, reaching the resonance condition for exothermic and endothermic TET processes, through thermal activation energy. The molecular dynamics simulations were compared with the static determination of the TET reaction coordinate (that defines the lowest activation energy structures for a given T_1_-S_0_ energy gap), finding a reliable agreement. Consequently, the TET reaction coordinate can be correctly determined via analysis of molecular dynamics simulations, including the coordinates responsible for the process.

With the aim of finding a more selective way, other than thermal energy, to modify the porphyrin structure in order to match a defined T_1_-S_0_ energy gap (thus maximizing a specific porphyrin-acceptor TET process), a substituent approach was proposed: dioxy-alkyl chains of different lengths were linked to two facing pyrrole units, thus generating a mechanochemical pushing force resulting in porphyrin sheet-bending. In this way, on the one hand, the structural deformation proposed by the TET reaction coordinate to reach highly exothermic resonance conditions (i.e., molecular oxygen as the acceptor) was achieved, as evidenced by a reduced T_1_-S_0_ energy gap as a function of the linker length. On the other hand, in-plane distortions were proposed by analysis of the TET reaction coordinate to reach less pronounced exothermic and even endothermic resonance conditions (i.e., carotenoids and rhodopsin, respectively). Although a substituent effect can hardly play a large role in planar modifications of porphyrin, we show that by simple methyl substitution on each pyrrole unit, a non-negligible increase in its T_1_-S_0_ energy gap can be indeed obtained.

Thus, we have firmly established the theoretical basis to design specific donors (or acceptors) capable of matching a desired singlet-triplet energy difference, in order to maximize the energy transfer process. A possible implementation was also proposed based on chemical substitution, mimicking the activation of the complex coordinates theoretically described. If applied to porphyrin as the donor, our results could play a role in the fields of photodynamic therapy and photosynthetic machineries.

## Supporting Information Available

S_0_-T_1_ energy gap and T_1_ activation energy for O_2_; porphyrin phosphorescence and oxygen absorption simulated spectra; details of the mechanochemical effects; analysis of the triplet state nature along the TET reaction coordinate; benchmark of different DFT functionals; and Cartesian coordinates (in Ångström) and energies of the T_1_ optimized structures (PDF)

## Notes

Moreover, the full code concerning the implementation of the molecular dynamics can be accessed at the following repository: https://github.com/resmol/dynamics
