# Supplementary material for: Thermal and Mechanochemical Tuning of the Porphyrin Singlet-Triplet Gap for Selective Energy Transfer Processes: A Molecular Dynamics Approach
Source: J Chem Theory Comput. 2021 Aug 5;17(9):5429–39. doi: 10.1021/acs.jctc.1c00291 (PMC8919258; doi:10.1021/acs.jctc.1c00291)
Supplement: Supplementary file 1 — ct1c00291_si_001.pdf [file ct1c00291_si_001.pdf]

Supporting Information

Thermal and Mechanochemical Tuning of Porphyrin Singlet-Triplet Gap for Selective Energy Transfer Processes: a Molecular Dynamics Approach

Felipe Zapata[1,‡,†], *Martina Nucci*[1,‡], *Obis Castaño*[1], *Marco Marazzi*[1,2,*], *Luis Manuel Frutos*[1,2,*]

[1] Departamento de Química Analítica, Química Física e Ingeniería Química, Universidad de Alcalá, Ctra. Madrid-Barcelona, Km 33.600, E28805, Alcalá de Henares, Madrid, Spain

[2] Instituto de Investigación Química ''Andrés M. del Rio'' (IQAR), Universidad de Alcalá, Ctra. Madrid-Barcelona, Km 33.600, E-28805, Alcalá de Henares, Madrid, Spain

Content

1. S_0-T_1 energy gap and T_1 activation energy for O_2
2. Porphyrin phosphorescence and oxygen absorption simulated spectra
3. Out-of-plane mechanochemical effects
4. Analysis of the triplet states nature along the triplet energy transfer reaction coordinate
5. Benchmark of different DFT functionals
6. Cartesian Coordinates (in Ångström) and energies of the T1 optimized structures

1. S_0-T_1 energy gap and T_1 activation energy for O_2

In order to calculate the T_1-S_0 energy difference as a function of the T_1 activation energy for O_2, as it was shown for porphyrin in Figure 2 of the main text, CASPT2(4,4) single point calculations for the singlet and the triplet states were performed for different bond lengths. The selected active space of 4 electrons in 4 molecular orbitals allows to describe the two π-bonds between the oxygen atoms. In Figures S1 and S2 is reported the polynomial fitting of the singlet and triplet energy as a function of the bond length of molecular oxygen, while in Figure S3 porphyrin and O_2 curves are shown.

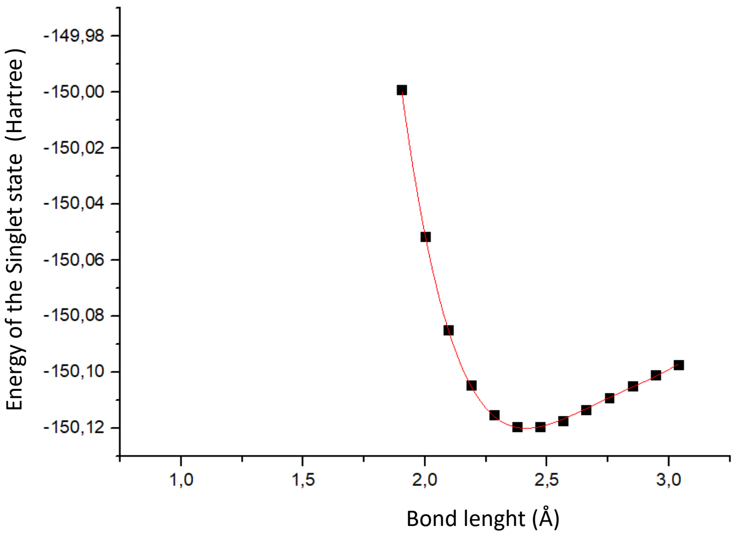

Figure S1. S_0 energy of O_2 as a function of the O-O bond length.

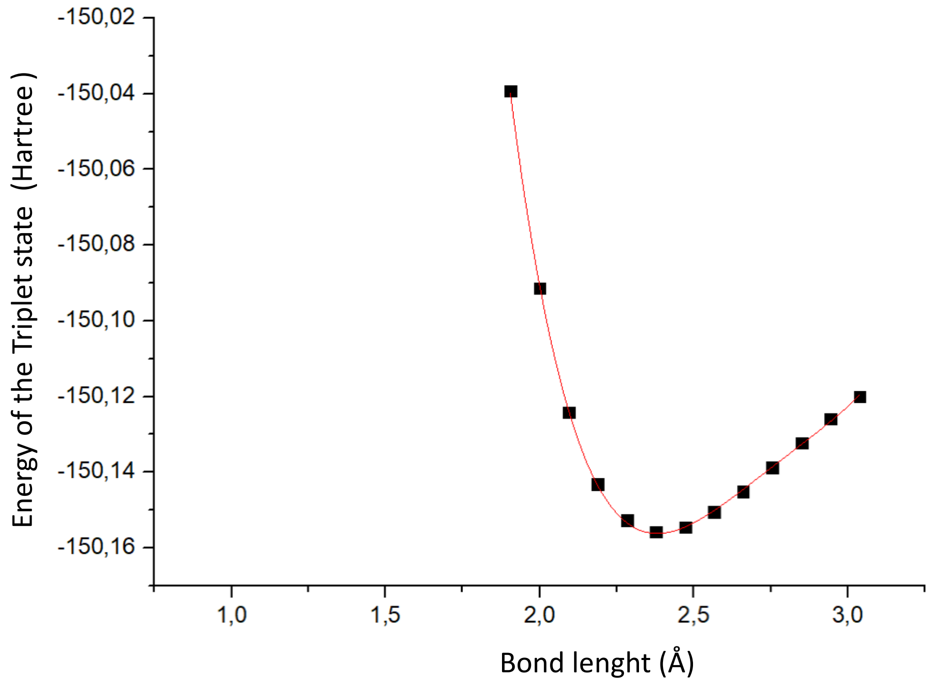

Figure S2. T_1 energy of O_2 as a function of the O-O bond length.

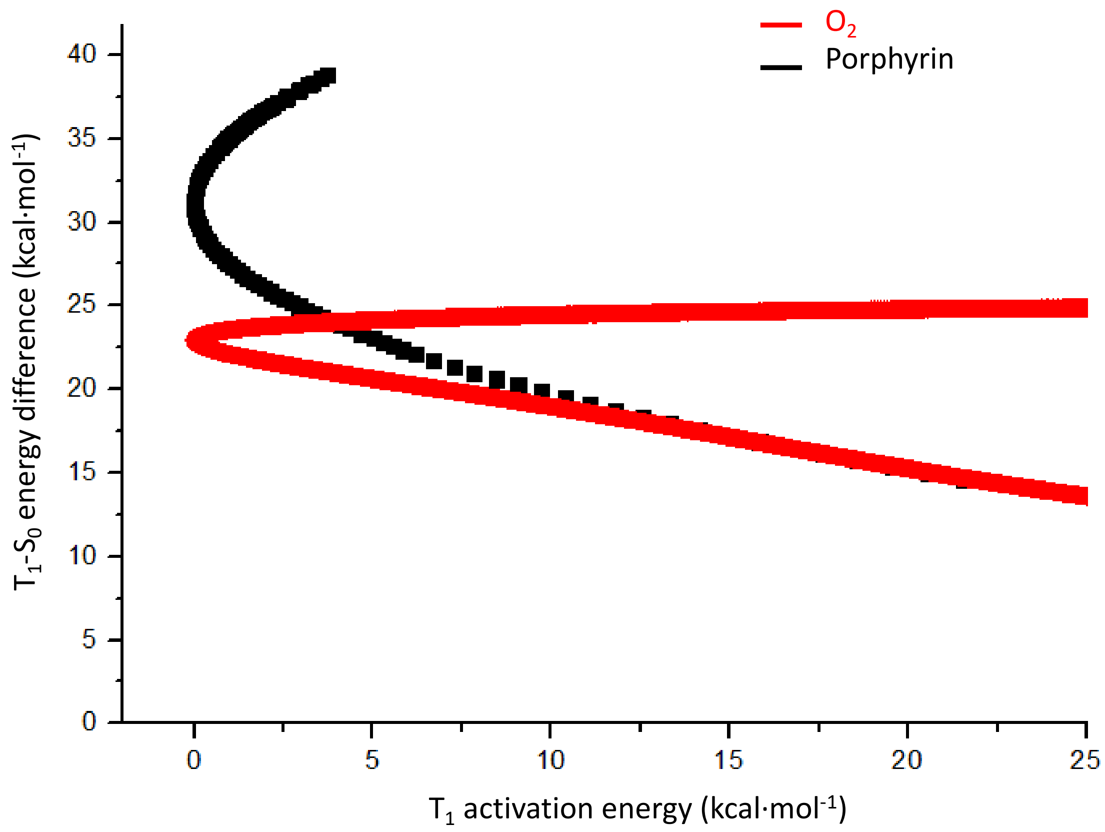

Figure S3. T_1-S_0 energy gap as a function of the activation energy on T_1, porphyrin in black and molecular oxygen in red.

2. Porphyrin phosphorescence and oxygen absorption simulated spectra

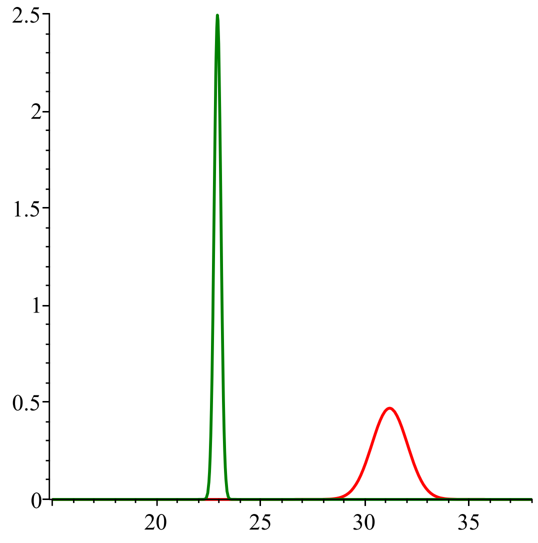

Figure S4. Simulated spectrum of porphyrin phosphorescence and molecular oxygen absorption at a temperature of 50K.

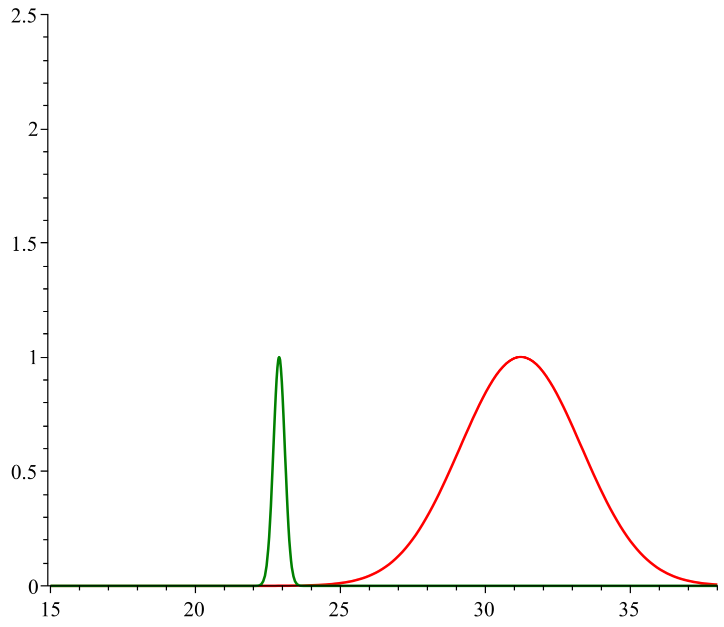

Figure S5. Simulated spectrum of porphyrin phosphorescence and molecular oxygen absorption at a temperature of 300K.

3. Mechanochemical effects

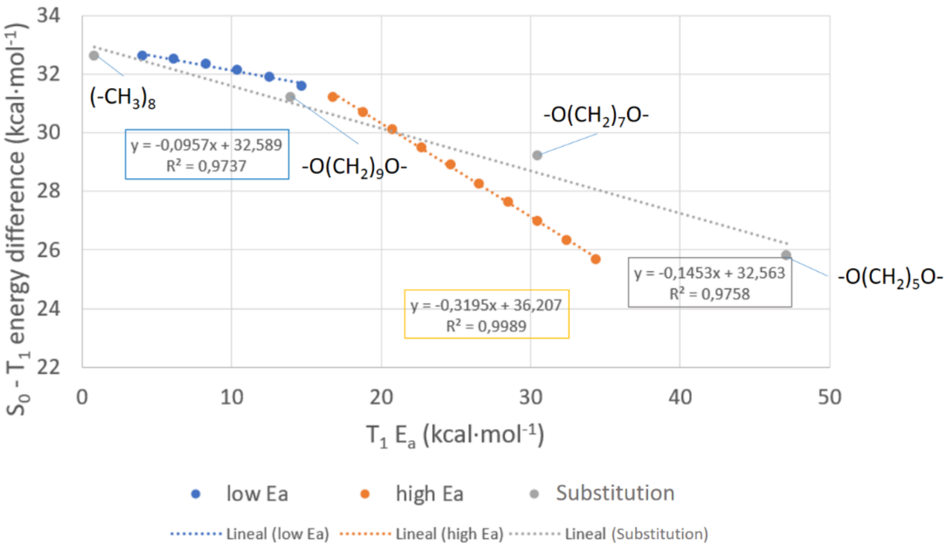

Figure S6. Values obtained by in-plane and out-of-plane distortions through porphyrin substitution at the porphyrin pyrrole rings (grey spots, see Figure 4 of the main text), and by performing a relaxed scan along the anchoring carbon atoms (blue and orange spots, depending on the values of the activation energy, T_1 E_a). The values of T_1 E_a are calculated by taking the optimized structures, deleting the substituents and replacing them with hydrogen atoms (i.e., re-establishing the porphyrin connectivity). The T_1 absolute energy value of the resulting structure is subtracted to the T_1 absolute energy value of unsubstituted optimized porphyrin, thus obtaining T_1 E_a. The linear regression parameters are shown as insets, following the color code. Nomenclature of the substituted compounds follows that of Figure 5 in the main text.

4. Analysis of the triplet states nature along the triplet energy transfer reaction coordinate

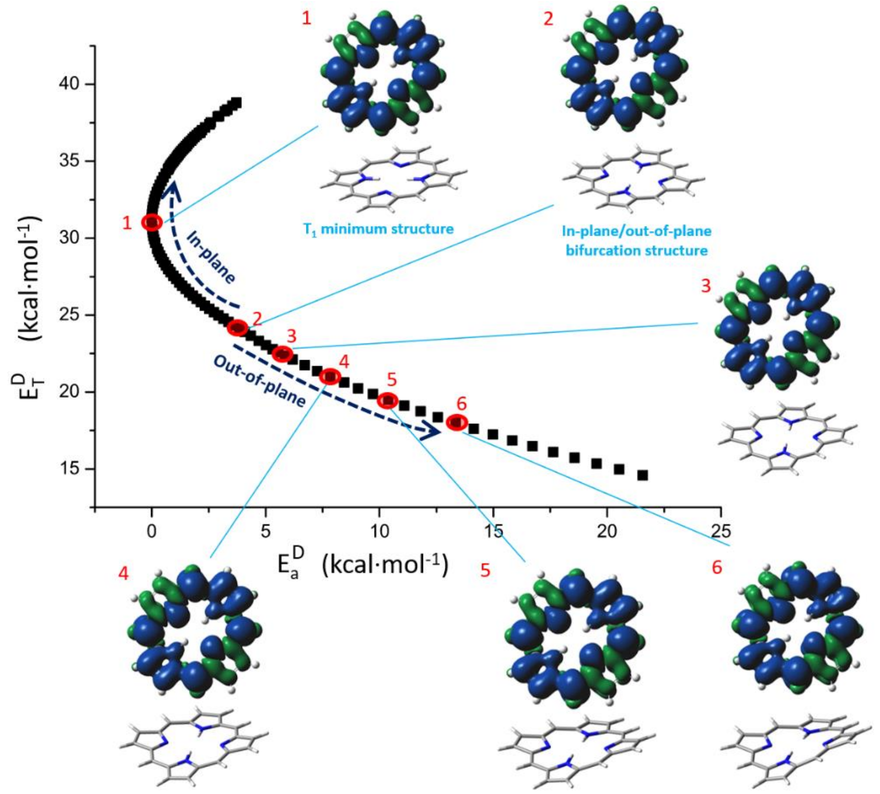

Figure S7. Spin density and geometry of six structures along the T_1 energy transfer reaction coordinate, as depicted in Figure 3 of the main text, characterized by increased out-of-plane bending.

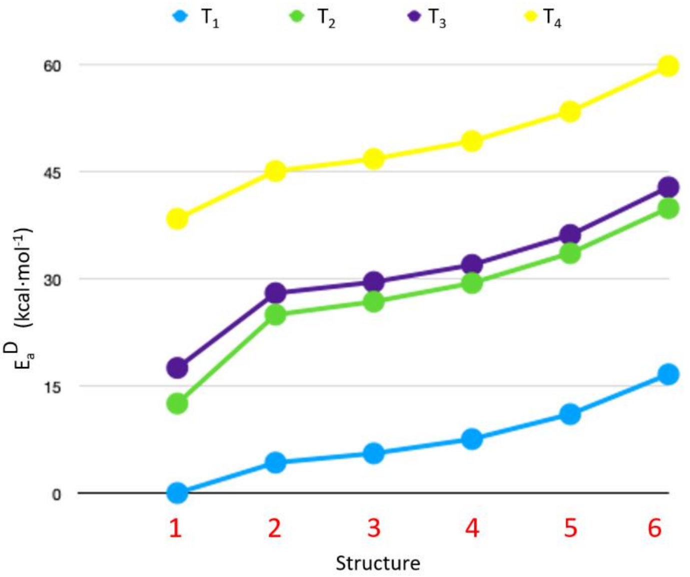

Figure S8. Energies of the electronic ground state triplet (T_1) and of the three lowest-energy triplet excited states (T_2, T_3 and T_4) of the six structures depicted in Figure S7.

5. Benchmark of different DFT functionals

Table S1. T_1 and S_0 absolute energies of T_1 optimized porphyrin. The relative T_1-S_0 gap is also given. Basis set: CC-pVDZ.

DFT Functional	T_1 energy (Hartree)	S_0 energy (Hartree)	T_1-S_0 gap (kcal/mol)
PBE0	-988.444023413	-988.492070128	30.149
M06-2X	-989,202531774	-989,250967302	30.939
CAM-B3LYP	-989,016113181	-989,054367136	24.004
B3LYP	-989,562522959	-989,612174261	31.156

The comparison among different functionals shows how the range-separated hybrid functional CAM-B3LYP underestimates the T_1-S_0 energy gap compared to the hybrid functionals PBE0, M06-2X and B3LYP.

6. Cartesian Coordinates (in Ångström) and energies of the T_1 optimized structures

Porphyrin
S_0 absolute energy = -989.612174261 Hartree
T_1 absolute energy = -989.562586959 Hartree

6	4.158848	-1.194522	0.000283
6	4.322703	0.200354	0.000274
6	2.773586	-1.458838	0.000179
7	2.126632	-0.248860	-0.000056
6	3.036271	0.778326	0.000176
6	2.691366	2.165846	0.000102
6	1.421207	2.733653	-0.000111
6	1.172550	4.177678	-0.000253
6	-0.174424	4.335347	-0.000322
6	-0.749828	2.987714	-0.000180
7	0.238867	2.033626	-0.000004
6	-2.116888	2.728919	-0.000165
6	-2.773708	1.458856	-0.000014
7	-2.126669	0.248867	-0.000199
6	-3.036401	-0.778304	-0.000012
6	-4.322768	-0.200375	0.000587
6	-4.158920	1.194552	0.000580
6	-2.691342	-2.165911	-0.000160
6	-1.421110	-2.733608	-0.000179
7	-0.238749	-2.033636	-0.000007
6	0.749919	-2.987780	-0.000107
6	0.174434	-4.335363	-0.000260
6	-1.172539	-4.177673	-0.000325
6	2.116932	-2.728861	0.000106
1	4.938390	-1.952616	0.000295

1	5.256679	0.757188	0.000281
1	1.116458	-0.130146	0.000186
1	3.539352	2.854402	0.000229
1	-0.745331	5.262786	-0.000471
1	-2.782501	3.595017	-0.000236
1	-1.116506	0.130151	-0.000216
1	-5.256695	-0.757280	0.000891
1	-4.938408	1.952693	0.000878
1	-3.539283	-2.854526	-0.000227
1	0.745345	-5.262834	-0.000362
1	2.782600	-3.594914	0.000233
1	1.942278	4.948164	-0.000347
1	-1.942258	-4.948126	-0.000476

Porphyrin $(-CH_3)_8$

S_0 absolute energy = -1304.15965761 Hartree

T_1 absolute energy =-1304.10846639 Hartree

6	-4.280827	0.703900	0.000256
6	-4.279898	-0.710251	0.000313
6	-2.924412	-1.125952	0.000243
6	-2.925994	1.121397	0.000113
7	-2.141778	-0.001667	0.000102
1	-1.125144	-0.000962	0.000071
6	-5.465083	1.625681	0.000342
6	-5.462958	-1.633544	0.000416
6	-2.418941	-2.461767	0.000193
6	-1.094123	-2.884962	0.000043
1	-3.182363	-3.241564	0.000214
6	-0.683339	-4.300689	-0.000077
6	0.680570	-4.299311	-0.000266
6	1.089010	-2.882244	-0.000258
7	-0.003768	-2.049952	-0.000030
6	-1.628159	-5.462689	-0.000165
6	1.627082	-5.459936	-0.000541
6	2.413217	-2.459793	-0.000387
6	2.925275	-1.124360	-0.000208
1	3.172352	-3.243252	-0.000598
6	4.275910	-0.705167	-0.000020
6	4.274841	0.711471	0.000062
6	2.923533	1.128643	-0.000030
7	2.140665	0.001504	-0.000166
6	5.497892	-1.572378	0.000276
6	5.495365	1.580724	0.001288
1	1.124493	0.000700	-0.000418
6	2.409553	2.463130	-0.000063
6	1.084486	2.883696	-0.000105
1	3.167542	3.247689	0.000041
7	-0.006786	2.049951	0.000025
6	0.674142	4.300186	-0.000269
6	-0.689778	4.299790	-0.000283
6	-1.098648	2.883535	-0.000060

6	1.619133	5.462033	-0.000450
6	-1.636098	5.460588	-0.000540
6	-2.422555	2.458239	-0.000022
1	-3.187287	3.236760	-0.000160
1	1.092942	-6.420917	0.000339
1	2.285918	-5.446819	0.885064
1	2.284389	-5.447620	-0.887306
1	-2.287668	-5.449634	0.884914
1	-1.092752	-6.422957	0.001775
1	-2.284985	-5.451687	-0.887292
1	-6.409261	-1.074179	0.000926
1	-5.473755	-2.290404	0.887285
1	-5.474367	-2.289856	-0.886856
1	-5.476559	2.282937	-0.886219
1	-5.477538	2.281559	0.887921
1	-6.410634	1.065076	-0.000626
1	-1.101940	6.421569	0.000239
1	-2.294897	5.447337	0.885070
1	-2.293581	5.448026	-0.887151
1	1.083721	6.422286	-0.000425
1	2.277111	5.450160	-0.886719
1	2.277360	5.450247	0.885635
1	5.247119	2.651482	-0.014101
1	6.135742	1.379867	-0.875629
1	6.119443	1.401499	0.894683
1	5.251424	-2.643652	-0.000619
1	6.129245	-1.381745	0.886063
1	6.130424	-1.380629	-0.884444

Porphyrin -O(-CH_2)$_9$O-
S_0 absolute energy = -1492.62416036 Hartree
T_1 absolute energy =-1492.57523699 Hartree

6	0.299704	4.705260	-0.731894
6	1.637699	4.293219	-0.660315
6	-0.492834	3.544448	-0.929383
7	0.374384	2.484615	-1.045331
6	1.664487	2.893751	-0.837277
6	2.752775	1.978859	-0.635441
6	2.779827	0.629162	-0.883398
6	3.866852	-0.252921	-0.433213
6	3.647302	-1.477102	-0.990890
6	2.356370	-1.359888	-1.674274
7	1.835257	-0.115554	-1.584625
6	1.672341	-2.466673	-2.243318
6	0.263903	-2.576162	-2.348411
7	-0.593205	-1.585060	-1.932885
6	-1.872134	-2.066135	-1.829178
6	-1.852934	-3.405575	-2.271647
6	-0.529838	-3.723454	-2.609392
6	-2.932119	-1.345623	-1.181420
6	-2.951621	-0.023892	-0.810133

7	-2.035730	0.961411	-1.172347
6	-2.546450	2.118778	-0.691573
6	-3.792613	1.912411	0.047785
6	-3.996184	0.566315	0.037596
6	-1.889953	3.374699	-0.777289
1	-0.089852	5.712687	-0.606478
1	2.511209	4.914607	-0.477279
1	0.061389	1.526364	-1.183848
1	3.642675	2.396822	-0.159154
1	4.212685	-2.396776	-0.857048
1	2.247723	-3.372013	-2.445591
1	-0.280947	-0.648887	-1.685133
1	-2.719608	-4.061467	-2.309179
1	-0.147441	-4.680904	-2.955075
1	-3.803877	-1.937293	-0.892310
1	-4.351486	2.674912	0.586449
1	-2.467298	4.267971	-0.532018
8	4.759366	0.180715	0.490881
8	-4.880874	-0.191008	0.743109
6	1.305545	-0.527227	3.263467
6	2.674875	-0.028617	2.751191
6	3.659623	-1.135484	2.336732
1	1.448025	-1.151730	4.166275
1	3.143725	0.597645	3.531777
1	2.500145	0.638598	1.891922
1	3.972803	-1.715950	3.224856
6	-2.021419	-0.615087	2.455389
6	0.488378	-1.320358	2.228606
1	0.727751	0.354511	3.593443
1	-1.890516	0.193533	3.197404
1	-1.872800	-0.151201	1.468493
1	0.449506	-0.755062	1.278880
6	-0.949153	-1.706577	2.638349
1	-1.250029	-2.568941	2.016904
1	-0.956457	-2.075158	3.681845
1	1.029006	-2.253013	1.994162
6	4.951386	-0.659779	1.657523
1	3.157095	-1.852779	1.670430
1	5.544577	-0.024136	2.332109
1	5.571903	-1.531628	1.381400
6	-3.452169	-1.182003	2.560893
6	-4.621176	-0.264983	2.170860
1	-3.632706	-1.502754	3.604175
1	-3.532987	-2.098779	1.949398
1	-4.481727	0.756834	2.566412
1	-5.558215	-0.667721	2.581505

Porphyrin -O(-CH_2)$_7$O-
S_0 absolute energy = -1413.96650414 Hartree
T_1 absolute energy =-1413.92075067 Hartree

6	-1.409577	-4.376165	-1.033330

6	-2.633213	-3.763681	-0.882922
6	-0.417918	-3.351495	-1.192023
7	-1.087227	-2.152609	-1.220421
6	-2.426897	-2.331977	-0.940325
6	-3.305254	-1.325934	-0.596646
6	-2.994096	0.094886	-0.525937
6	-3.591660	0.996610	0.464650
6	-3.279645	2.278961	0.077764
6	-2.340800	2.119807	-1.002557
7	-2.180965	0.757227	-1.342917
6	-1.505822	3.130404	-1.479943
6	-0.169365	2.957088	-1.878605
7	0.509220	1.760669	-1.806570
6	1.873586	1.974314	-1.768857
6	2.061652	3.390807	-2.010638
6	0.822413	3.980164	-2.073105
6	2.820472	1.053364	-1.370675
6	2.584553	-0.324204	-0.987396
7	1.563917	-1.080848	-1.391419
6	1.860675	-2.384827	-0.941147
6	3.102247	-2.405900	-0.209356
6	3.506143	-1.096626	-0.145583
6	0.978446	-3.455358	-1.040145
1	-1.193466	-5.441793	-0.993893
1	-3.596119	-4.236324	-0.703217
1	-0.601733	-1.262850	-1.289568
1	-4.291991	-1.625990	-0.240626
1	-3.512187	3.208803	0.592687
1	-1.826880	4.164934	-1.335105
1	0.050713	0.869168	-1.647496
1	3.033140	3.876185	-2.071511
1	0.597265	5.038309	-2.189344
1	3.843818	1.414134	-1.255501
1	3.533782	-3.261745	0.305567
1	1.364083	-4.452160	-0.813606
8	-4.096597	0.543195	1.652989
8	4.457954	-0.539939	0.664126
6	-3.151715	0.728389	2.750418
6	-1.822450	-0.020391	2.538749
6	-0.601670	0.554873	3.288285
6	0.723319	0.216507	2.573251
6	1.978469	0.858772	3.195916
6	3.196694	0.939247	2.247583
6	3.986989	-0.369560	2.031331
1	-3.693794	0.378335	3.640873
1	-2.962237	1.810953	2.864443
1	-1.589130	0.031670	1.464655
1	-1.953255	-1.091513	2.767332
1	-0.576664	0.204301	4.335938
1	-0.693063	1.656267	3.336644
1	0.636513	0.568101	1.528944
1	0.840917	-0.880384	2.508092

1	2.250896	0.340622	4.134585
1	1.725355	1.892702	3.491963
1	3.890501	1.713030	2.616465
1	2.846189	1.296384	1.266202
1	3.379326	-1.250387	2.299021
1	4.899093	-0.386315	2.646275

Porphyrin -O(-CH_2)$_5$O-
S_0 absolute energy = -1335.30312328 Hartree
T_1 absolute energy =-1335.26279902 Hartree

6	-0.821759	-4.284818	-1.453481
6	-2.070384	-3.757440	-1.283922
6	0.129013	-3.191755	-1.469216
7	-0.614724	-2.031244	-1.465870
6	-1.946270	-2.307349	-1.196216
6	-2.877754	-1.420119	-0.725022
6	-2.685990	0.004908	-0.492044
6	-3.292178	0.740540	0.624760
6	-3.124849	2.081539	0.356381
6	-2.242862	2.118827	-0.780647
7	-1.987179	0.829360	-1.273633
6	-1.488629	3.248102	-1.161153
6	-0.128947	3.191547	-1.469370
7	0.614791	2.031051	-1.465881
6	1.946363	2.307190	-1.196365
6	2.070457	3.757271	-1.284251
6	0.821809	4.284617	-1.453796
6	2.877863	1.419988	-0.725159
6	2.686039	-0.005030	-0.492016
7	1.987336	-0.829546	-1.273640
6	2.242868	-2.118962	-0.780472
6	3.124635	-2.081599	0.356714
6	3.292034	-0.740573	0.624938
6	1.488672	-3.248286	-1.160980
1	-0.545892	-5.336502	-1.497544
1	-3.009678	-4.294413	-1.169833
1	-0.173701	-1.119550	-1.401304
1	-3.833004	-1.838419	-0.401579
1	-3.396366	2.926040	0.986434
1	-1.899098	4.239145	-0.954649
1	0.173753	1.119377	-1.401113
1	3.009755	4.294270	-1.170313
1	0.545931	5.336292	-1.498010
1	3.833189	1.838260	-0.401905
1	3.396055	-2.926018	0.986916
1	1.899144	-4.239309	-0.954381
8	-3.650943	0.128687	1.796827
8	3.650797	-0.128632	1.796999
6	2.625242	-0.243265	2.836050
6	1.234989	0.133104	2.313331
6	-0.000030	0.000104	3.236538

6	-1.234900	-0.131711	2.312991
6	-2.625451	0.243207	2.835975
1	2.974339	0.427283	3.635110
1	2.626541	-1.278694	3.224566
1	1.041537	-0.515011	1.445785
1	1.275221	1.162677	1.918066
1	-0.087411	0.881023	3.896817
1	0.087301	-0.881726	3.895611
1	-1.274688	-1.160671	1.916094
1	-1.041545	0.517880	1.446527
1	-2.627567	1.278307	3.225381
1	-2.974075	-0.428270	3.634465
